# Mission Alliance Community Engagement Project: Exploring the Impact of COVID-19 on Social Isolation, Loneliness, Mental Health and Wellbeing in Veterans

**DOI:** 10.1007/s10900-023-01314-5

**Published:** 2023-12-08

**Authors:** Beth A. Pratt, Cheryl A. Krause-Parello, Viann N. Nguyen-Feng, Nicholas A. Giordano, S. Basilia Basin, Alan L. Peterson, Patrick Walsh, Aaron Q. Siebert, Rigoberto Ruiz, David M. Kirkland, John Paul Nolan

**Affiliations:** 1 College of Nursing, Florida Atlantic University, 3200 College Drive, LA49 228B, Davie, Boca Raton, FL 33314 USA; 2 Department of Psychology, University of Minnesota Duluth, Duluth, MN USA; 3 School of Nursing, Emory University, Atlanta, GA USA; 4 Oregon Health and Science University, Portland, OR USA; 5 University of Texas Health Science Center at San Antonio, San Antonio, TX USA; 6 University of Rochester Medical Center, Rochester, NY USA; 7 Warrior Built Foundation, Lake Elsinore, CA USA; 8 San Diego, CA USA; 9 Grey Team, Boca Raton, FL USA; 10 El Dorado, AR USA

**Keywords:** Community engagement, Veterans, Posttraumatic stress disorder, PTSD, Patient-centered outcomes research

## Abstract

During the Coronavirus disease pandemic, many U.S. veterans with posttraumatic stress disorder (PTSD) experienced increased symptomology and worsened mental health and well-being due in part to social isolation and loneliness. The Mission Alliance project explored these ramifications and prioritized critical issues expressed by U.S. veterans and stakeholders (*N* = 182) during virtual regional meetings (*N* = 32). Field notes created specifically for this project were recorded and thematically analyzed. Emerging themes included: (1) social isolation: missed opportunities, collapsed social circles, work-life balance, fostering relationships, and evolving health care delivery; (2) loneliness: deteriorated mental health, suffered with PTSD together but alone, looked out for each other, ambivalence toward technology, and strained and broken systems; (3) mental health: sense of chaos, increased demand and decreased access, aggravation, implementation of tools, innovative solutions, fear and loss, and availability of resources; (4) wellbeing: sense of purpose, holistic perspective on well-being, recognition of balance, persisting stigma, redefined pressures, freedom to direct treatment, and reconnection and disconnection. A PTSD-related patient centered outcomes research (PCOR)/comparative effectiveness research (CER) agenda was developed from these themes. Establishment of a veteran and stakeholder network is suggested to support, facilitate, and promote the PTSD-related PCOR/CER agenda. Furthermore, enhancement of opportunities for veterans with PTSD and stakeholders to partner in PCOR/CER is required to develop and conduct projects that lead to PTSD-related comprehensive care of veterans affected by traumatic events with the potential to translate findings to other populations.

There are 17.4 million United States (US) military veterans, of which 1.7 million receive care at the Veterans Health Administration (VA) facilities for mental health conditions such as posttraumatic stress disorder (PTSD) [1, 2]. In the US adult population, PTSD has a lifetime prevalence rate of 6.8% [3]. In contrast, approximately 13.8% of Operation Iraqi Freedom/Operation Enduring Freedom veterans, 10.1% of Gulf War veterans, and up to 30.9% of Vietnam veterans have PTSD [3]. Veterans with PTSD were significantly affected by the catastrophic impact of the Coronavirus disease (COVID-19) pandemic [4, 5]. PTSD symptoms such as avoidance of people and places, sleep disturbances, and hypervigilance resurfaced and worsened due to the resultant social isolation and loneliness experienced during COVID-19. Veterans’ PTSD symptoms were also triggered by the spread of misinformation, fear of becoming infected, and the media’s reference of the COVID-19 pandemic as a war [6].

## Social Isolation and Loneliness

The health of veterans living with PTSD was greatly affected by the COVID-19 pandemic due to the social isolation and distancing measures implemented, social disparities, and pre-existing comorbidities that increased the risk for negative health outcomes [7]. Social isolation weakened veterans’ support systems and caused a greater incidence of loneliness, particularly for veterans with functional limitations, advanced age, and mental health challenges [8, 9]. Specifically, the COVID-19 pandemic had a profound psychological impact on veterans experiencing PTSD symptoms, affecting not only these veterans, but their families, who are often their primary source of support through mental health challenges [10].

## Mental Health and Well-Being

Veterans’ overall well-being declined due to adversities experienced throughout the COVID-19 pandemic including professional, financial, and personal complications, complex renegotiations of partner and family roles, and the lack of established social support networks [11]. The resultant isolation from family, friends, and peers increased veterans’ reluctance to receive aid and support, and consequently decreased their access to proper health care, thereby affecting mental health and well-being. Even after social isolation and distancing measures were lifted, veterans continued to experience persistent negative mental health outcomes and decreased well-being [12, 13].

## Community-Based Approach

Historically, veterans with PTSD have been excluded from providing meaningful input on healthcare interventions and preferences for treatment options that align with their unique experiences. In addition, veterans have been reluctant to engage in the research process due to lack of trust and perceived alienation from the civilian population. This reluctance and lack of participation in community engagement activities has contributed to existing gaps in PTSD-related patient-centered outcomes research (PCOR) driven by patients’ and stakeholders’ concerns and focused on their preferred outcomes [14] and comparative effectiveness research (CER) of two or more medical treatments, services, or health practices to assist patients and stakeholders make better healthcare decisions [15]. Their non-engagement in PCOR/CER has exacerbated an already existing barrier to quality health care for veterans.

To address this issue, veteran partners assisted in identifying the needs of veterans with PTSD and crafting a community engagement project, Mission Alliance, and its objectives. A Mission Alliance Veteran Unit Leader, Retired US Army Specialist John Paul Nolan, Junior, emphasized:

As a community, veterans have difficulty integrating under the best of circumstances. COVID-19 acutely intensified this situation. The very nature of veterans with PTSD is to avoid everything at all costs. The COVID-19 lockdown allowed every veteran with PTSD (diagnosed or not) to shut down completely. Unfortunately, veterans with mental health issues are likely to self-medicate first. During COVID-19, substance abuse skyrocketed, and many veterans who had made gains in this area lost years of substance-free living, which negatively affected their families. There was an intense loss of confidence in healthcare systems, VA and non-VA, and confusion about vaccines. Although telehealth made great strides, there was a shortage of personnel and equipment to deal with the high demand for services during COVID-19. These intense systemic amplifications will persist, and it is crucial to show us compassion and respect to better assess our mental health and well-being and to aid where able.

His impressions demonstrated a clear obligation to engage veterans with PTSD in order to fully understand their comprehensive healthcare needs that could lead to promotion of improved mental health and well-being during times of uncertainty and stress such as experienced during the COVID-19 pandemic.

The main objective of the Mission Alliance community engagement project was to develop veteran-driven PTSD-related research priorities related to COVID-19 in full partnership with veterans and key stakeholders (see Table 1). Mission Alliance unit members had the opportunity to develop and strengthen the relationship with veterans who have PTSD and key community stakeholders affected by or concerned with PTSD and to provide a platform for them to have a voice in solutions for addressing PTSD treatment and clarifying desired outcomes of treatment. Their participation in Mission Alliance’s neutral and engaging virtual environment furthered understanding of COVID-19’s impact on social isolation, loneliness, mental health, and well-being; provided opinions on emerging PTSD-related PCOR and CER needs; and described facilitators and barriers to participation in PCOR and CER. These discussions were the building blocks of the prioritized veteran-driven COVID-19 PTSD-related PCOR and CER agenda.

**Table 1:** List of the veteran-driven COVID-19 PTSD-related PCOR/CER priorities

Social isolation	1. What are the potential benefits and harms of using online platforms vs. in-person meetings to reduce social isolation for veterans with PTSD?
	2. Are there differences in healthcare outcomes, including social isolation, for veterans with PTSD who receive care via telehealth vs. in-person appointments?
	3. What are preventative measures that healthcare providers and veterans with PTSD can use to mitigate the effects of social isolation?
	4. What beneficial vs. harmful factors (e.g., perceived sense of control) moderate levels of social isolation among veterans with PTSD versus civilians with PTSD?
	5. Which platforms and formats foster higher engagement among veterans with PTSD? Additionally, which are linked to improved well-being and mitigates social isolation?
	6. What are the benefits and harms of clinician-led versus peer-to-peer support regarding social isolation for veterans with PTSD?
	7. What workplace policies and COVID-related regulations are linked to improved quality of life and mitigate social isolation for veterans with PTSD?
Loneliness	1. How do interpersonal relationships and loneliness relate to substance use and mental health outcomes for veterans with PTSD?
	2. What interpersonal responses including vocational and recreational activities impact loneliness outcomes for veterans with PTSD?
	3. What frequency and types of engagement by support systems improve loneliness and quality of life for veterans with PTSD?
	4. Which forms of communication are more conducive to mitigate loneliness for older adult veterans with PTSD who face barriers to utilizing technology?
	5. What are the benefits and harms of clinician-led versus peer-to-peer support regarding loneliness for veterans with PTSD?
	6. What is the effectiveness of civilian patient navigators compared to VA/DoD veteran patient navigators for veterans with PTSD who experience loneliness?
	7. What are effective and efficient methods for primary health care providers to screen for loneliness in veterans with PTSD?
Mental health	1. What routines, structures, internal factors improve mental health in veterans with PTSD?
	2. What integrative interventions improve mental health in veterans with PTSD?
	3. What complementary/integrative interventions in conjunction with provider-driven evidenced-based treatments affect mental health among veterans with PTSD?
	4. What coping strategies mediate the relationship between perceived social support and improved mental health for veterans with PTSD during transitions?
	5. What platforms and formats connect veterans with PTSD, across different geographic locations, to credible resources that impact mental health?
	6. What behavioral self-assessment tools/training enhance self-awareness and acceptance of varying viewpoints to impact mental health in veterans with PTSD?
	7. What mechanisms or means of transportation are most effective to facilitate access to and attendance to mental health care appointments?
Well-being	1. What vocational factors related to purpose impact well-being in veterans with PTSD?
	2. What veteran-delivered complementary/integrative interventions compared to provider-delivered interventions impact well-being in veterans with PTSD?
	3. How do individual compared to dyadic psychotherapies affect well-being in veterans with PTSD?
	4. What factors facilitated or hindered coping skills during the COVD-19 pandemic to maintain well-being in veterans with PTSD symptoms?
	5. What factors related to autonomy impacted well-being in veterans with PTSD during COVID-19?
	6. How do you support and enhance training for veterans who are in leadership positions to enhance well-being in veterans with PTSD?
	7. How do health systems, work places and community organizations design spaces that enable veterans from various points in their transitional period to enhance social engagement and sense of well-being?

## Method

The university Institutional Review Board (IRB) deemed the Mission Alliance community engagement project non-human subjects research (#1825771). In the initial stages of the proposal, the project lead and co-lead, who are academic researchers, created four regional units in the Midwest, Northeast, South, and West regions of the US each led by a military veteran and an academic researcher. The entire Mission Alliance team consisted of the project lead, co-lead, four academic researchers, four US military veterans, and two US military veteran PTSD expert consultants who worked together to build and strengthen key community stakeholder relationships.

The project officially began in November 2021 and ended in April 2023. Prior to engagement with the larger veteran and key stakeholder community, Mission Alliance team members completed training from PCORI (https://www.pcori.org/engagement/research-fundamentals#content-6876) and Operation PCOR (https://www.operationpcor.com/). They also held quarterly team meetings to create four field note templates focused on the topics of social isolation, loneliness, mental health and well-being as related to COVID-19 that promoted facilitation of the veteran and community stakeholder virtual regional meetings and two evaluation tools to be distributed to attendees after meetings.

The Mission Alliance Veteran Unit Leaders and academic researchers recruited local and regional veterans with PTSD and key community stakeholders via social media, flyers, and word of mouth to attend meetings that were held on a video-conferencing platform for up to 1 h. The Veteran Unit Leaders facilitated these meetings and encouraged veterans and stakeholders to express their views related to the impact of COVID-19 on social isolation, loneliness, mental health, and well-being. The academic researchers took field notes based on the templates, summarized the topics discussed in the unit meetings, and sent their anonymized summaries from each virtual unit meeting to the Project Lead. The Veteran Unit Leaders and academic researchers aided in logistical planning of monthly meetings, execution of deliverables, and development of the veteran-driven research priorities. Additionally, Veteran Unit Leaders assisted the Project Leads in interpreting veterans’ expressed views and needs.

The Project Lead aggregated field notes from the regional meetings for thematic analysis [16]. The initial step in analysis involved reading and re-reading the notes to become familiar with the content and to generate preliminary ideas for codes. After familiarization, initial codes were assigned to describe meaningful pieces of data obtained from the meetings, and the data were then grouped into the most relevant codes. Upon review of the codes and associated data, broader themes were developed to interpret the data. An iterative process was implemented to move back and forth between the broad themes, codes, and data to create the final set of themes and ensure that they provided adequate representation of the meetings’ content in a coherent and distinctive manner. The essence of each theme was identified and provided the basis for creation of the PCOR/CER questions created during the Mission Alliance retreat at the end of the project.

## Results

Regional teams held 32 virtual meetings across the US with a total of 182 attendees. The meetings included 139 veterans who served during the Korean War (*n* = 2), Vietnam War (*n* = 9), Desert Shield/Desert Storm (*n* = 35), and the Global War on Terror (*n* = 90). Three veterans did not disclose their period of military service. There were an additional 43 key community stakeholders including family members, veteran support organization members, policymakers, PTSD therapists, and other health care providers in attendance.

### Project Deliverables

The Mission Alliance team created several tools throughout the project period, including: (a) four field guide templates with open-ended questions related to social isolation, loneliness, mental health, and well-being as experienced during COVID-19; (b) three evaluation forms; (c) two e-Magazines to disseminate meeting highlights; (d) the prioritized COVID-19 PTSD-related PCOR/CER priorities connected with social isolation, loneliness, mental health, and well-being; and (e) the Mission Alliance Handbook which is available for public use (https://nursing.fau.edu/documents/cpaww/Mission-Alliance-Handbook-Final-2023.pdf). The team disseminated findings to 21 Veterans and stakeholders during the Mission Alliance National Virtual Convening that was held on March 10, 2023.

### Themes

The analysis revealed themes related to the topics of social isolation, loneliness, mental health, and well-being as experienced during the COVID-19 pandemic. These findings shaped the veteran-driven PTSD-related PCOR/CER agenda.

#### Social Isolation

Social isolation, the perceived and/or actual lack of social connections, has been linked to poor health outcomes as well as decreased physical and mental well-being among veterans [9, 17]. Understanding the impact of social isolation and potential means by which to assess and address it among veterans during the COVID-19 pandemic was a priority among attendees of the virtual regional meetings. Notably, five key themes related to social isolation emerged from these discussions: missed opportunities, collapsed social circles, work-life balance, fostering relationships, and evolving health care delivery.

Veterans expressed missing opportunities to engage with others as a key concern during the pandemic in response to social distancing requirements. Opportunities to meet in person with friends, family, and other veterans for recreational, occupational, and other social activities were perceived as lacking during the pandemic by attendees and contributed towards feeling isolated. Further, attendees sensed that their social circle collapsed during this time period. Some felt that their families were “torn apart” due to different viewpoints on COVID-related mandates and recommendations. Communication with others felt protracted and difficult to maintain compared to the comradery experienced from in-person meet ups and social gatherings. Attendees reported feeling they had to cultivate a limited number of close relationships, through more frequent and personal communication, during the pandemic than prior to it to mitigate feeling isolated.

Many veterans who attended the virtual regional meetings worked during the pandemic and felt an imbalance in their work/life commitments. For instance, individuals working remotely at home during the pandemic felt constantly tethered to their work communications while others working in essential in-person occupations felt overloaded with additional stressors of work obligations, additional shifts, and exposing others, including those they lived with, to the spread of COVID-19. Much like the experiences of the US adult general population [18], veterans shared that the evolving delivery of health care, specifically mental health therapies, were difficult to keep up with as group therapy sessions and individual therapy appointments moved to an online delivery. This initial transition was noted as somewhat challenging by attendees at the beginning of the pandemic, but later felt accustomed to the flexibility offered by both online therapies and even other health care providers who would see veterans via telehealth.

#### Loneliness

Loneliness is defined as the subjective social experience one would like to have as compared to one’s actual social network [9]. During the virtual regional meetings, attendees described aspects of loneliness related to their social network changes and the impact this had on their lives in relation to their experience with PTSD. Five key themes focused on loneliness emerged from the analyses of the field notes: deteriorated mental health, suffered with PTSD together but alone, looked out for each other, ambivalence toward technology, and strained and broken systems.

Attendees shared that the changes in their social connections due to COVID-19 guidelines and recommendations for physical distancing, masking, and vaccinating wreaked havoc on their mental health. For some veterans, these recommendations were triggers for their PTSD symptoms, resulting in family and friends rejecting the veteran, thus creating an increased sense of loneliness. The attendees described that they felt abandoned and lonely when their veteran’s centers closed, and the VA was less available for them to access care and connect with other veterans. The places and groups that the veterans once visited to get support for PTSD symptom management were no longer available, and they had to suffer alone.

Some attendees experienced suicide ideation, increased alcohol and substance use, and feelings of worthlessness. However, the lack of onsite/in-person connection led some veterans to leverage technology to reconnect and look out for one another. In-person appointments or meetings evolved into virtual meetings, and veterans utilized text messaging and virtual platforms to stay connected to their “Battle Buddies.” These virtual connections often rekindled relationships that were otherwise lost with time. Other attendees, however, had conflicting feelings toward technology. Attendees who were older had a more challenging time accessing and using the internet and social media, especially those in rural areas. Some veterans felt that virtual groups were “not the same,” and they found it more difficult to build trust with others and make new connections online.

Further, attendees expressed that the healthcare system was strained by the vast number of people who attempted to access services and broken by the inability to accommodate these large numbers. They believed that health care providers spread “fear and misinformation” related to the pandemic, which fueled their sense of hopelessness. Attendees found it difficult to access and receive adequate mental health care including the ability of healthcare providers to identify loneliness. Unfortunately, many reported that the relationships with their healthcare providers eroded over time resulting in decreased motivation to seek help.

#### Mental Health

Seven key themes related to mental health emerged from the analyses of the meetings’ field notes: sense of chaos, increased demand and decreased access, aggravation, implementation of tools, innovative solutions, fear and loss, and availability of resources. Attendees described a sense of feeling out of control, particularly in relation to inescapable “chatter” in their minds. They also felt an inability to manage everyday activities and stressors alone, especially in combination with pride in “handling [things] on [their] own” and apprehension in seeking support. Further, attendees noted an increased volume of need for mental health services coupled with inequality in access to care that led some attendees to reject any method of dealing with mental health issues. Aggravation appeared to stem from senses of re-traumatization and betrayal with the healthcare system and government.

Veterans implemented supportive tools such as implementing daily structure and routine, practicing optimism, walking away from arguments, talking to family, checking in with other veterans, taking leadership roles in veteran communities, and joining veteran groups. Innovative tools and solutions included animal therapy (e.g., horses, dogs), art therapy, workplace whole-health initiatives, recreational leagues (e.g., chess, frisbee), meditation and mindfulness activities, and seeking guest speakers in community events and healthcare provider support at home. Nonetheless, feelings of fear and loss arose that impacted mental health, including experiencing constantly changing pandemic mandates, having family and friends die from COVID, and losing support and regular communication from family, such as spouses invalidating veterans by saying that the veteran “shouldn’t have a problem”. Resources appeared decentralized with limited access to credible sources of information, especially on social media.

#### Well-Being

The seven key themes related to well-being that emerged from the field notes included: sense of purpose, holistic perspective on well-being, recognition of balance, persisting stigma, redefined pressures, freedom to direct treatment, and reconnection and disconnection. Attendees reported a sense of purpose when helping others navigate through life and achieve goals as well as using their own perceived gifts, talents, and skills in a way that was deemed useful to others. They took particular pride in teaching veterans how to perform new skills and informing others about the veteran experience and PTSD. Holistic perspectives on well-being included doing things in moderation, tracking stress and anxiety through methods such as journaling, practicing gratitude for life, and building a sense of community.

Attendees described increased ability to self-examine and notice diminished balance in various arenas, including family and children, socializing, public health, and learning to “walk in love” and “take things lightly” rather than fighting. Yet, there were persisting stigmas and “pity,” such as workplaces and families using veterans’ PTSD status to discriminate and shame them in relation to accommodations (“they are hard to deal with”) as well as societal expectations that veterans should not be affected, “You should be able to handle this, you’ve been through worse,” with attention deserved only if “famous”. Pressures became redefined, extending newly to technology and its limited access, inability to schedule time off to compensate for extra time needed for COVID recovery, inability to see nonverbal cues due to face masks, and increased demands of families, children at home, and long working hours. Nonetheless, attendees discussed a sense of freedom to direct treatment given the variety of options through virtual therapy and mobile apps as well as complementary and integrative approaches such as meditation, music therapy, dance therapy, herbal medication, tapping, and acupuncture.

## Discussion

This community engagement project uncovered the effects of social isolation, loneliness, mental health, and well-being for veterans with PTSD during the COVID-19 pandemic. Due to the preexisting disadvantages and comorbidities veterans face such as low socioeconomic status and untreated mental health conditions, they were especially vulnerable to experiencing profound negative impacts on their health and well-being [19]. This project provided veterans with PTSD and key community stakeholders an opportunity to provide meaningful input on interventions and treatment preferences for PTSD symptom management that align with their unique experiences and priorities. Even though there is oftentimes a reluctance on the part of veterans with PTSD to engage in the research process due to lack of trust and perceived alienation from the civilian population, this project was successful in engaging the veteran community.

Veterans have an increased lifetime risk of developing PTSD symptoms, and the results of this project supported that many veterans with PTSD were significantly affected by the catastrophic impact of the COVID-19 pandemic [4, 11, 13]. Veterans who were socially isolated had increased feelings of loneliness that impacted their mental health and well-being. The social isolation and uncertainty created by the COVID-19 pandemic was devastating for many veterans with PTSD who are at even greater risk for short-term and long-term mental health sequalae. The psychological impact of the COVID-19 pandemic on veterans’ PTSD symptoms affected not only themselves, but their families and communities. The themes that emerged from the regional unit meetings indicate the importance of future research dedicated to understanding and addressing social isolation, loneliness, mental health, and well-being among veterans with PTSD.

## Conclusions

The Mission Alliance project engaged Veterans and key community stakeholders throughout the US during virtual meetings to increase their understanding of PCOR/CER and provide space for them to share experiences and opinions on critical PCOR/CER needs. Our team suggests that veteran and stakeholder networks be established for continued community support, facilitation, and promotion of the project’s COVID-19 PTSD-related PCOR/CER priorities. Furthermore, Veteran and stakeholder opportunities as PCOR/CER partners need to be enhanced when developing and conducting PCOR/CER projects. This may lead to PTSD-related comprehensive care of veterans affected by COVID-19, potentially translating findings to other populations and traumatic events.

## Data Availability

The data that support the findings of this study are available from the corresponding author, BAP, upon reasonable request.
